# The Applicability of the International Staging System in Chinese Patients with Multiple Myeloma Receiving Bortezomib or Thalidomide-Based Regimens as Induction Therapy: A Multicenter Analysis

**DOI:** 10.1155/2015/856704

**Published:** 2015-11-08

**Authors:** Jing Lu, Jin Lu, Aijun Liu, Weijun Fu, Juan Du, Xiaojun Huang, Wenming Chen, Jian Hou

**Affiliations:** ^1^Department of Hematology, The Myeloma and Lymphoma Center, Changzheng Hospital, The Second Military Medical University, 415 Fengyang Road, Shanghai 200003, China; ^2^Peking University People's Hospital and Peking University Institute of Hematology, No. 11 Xizhimen South Street, Beijing 100044, China; ^3^Department of Hematology, Beijing Chaoyang Hospital, Capital Medical University, 8 Workers Stadium South Road, Beijing 100020, China

## Abstract

The International Staging System (ISS) is the most important prognostic system for multiple myeloma (MM). It was identified in the era of conventional agents. The outcome of MM has significantly changed by novel agents. Thus the applicability of ISS system in the era of novel agents in Chinese patients needs to be demonstrated. We retrospectively analyzed the clinical outcomes and prognostic significance of ISS system in 1016 patients with newly diagnosed multiple myeloma in Chinese patients between 2008 and 2012, who received bortezomib- or thalidomide-based regimens as first-line therapy. The median overall survival (OS) of patients for ISS stages I/II/III was not reached/55.4 months/41.7 months (*p* < 0.001), and the median progression-free survival (PFS) was 30/29.5/25 months (*p* = 0.072), respectively. Statistically significant difference in survival was confirmed among three ISS stages in thalidomide-based group, but not between ISS stages I and II in bortezomib-based group. These findings suggest that ISS system can predict the survival in the era of novel agents in Chinese MM patients, and bortezomib may have the potential to partially overcome adverse effect of risk factors on survival, especially in higher stage of ISS system.

## 1. Introduction

Multiple myeloma (MM) is the second most common hematological malignancy, accounting for 10% of all neoplastic hematologic disorders. It is characterized by significant heterogeneities in clinical manifestations and prognosis. The median overall survival (OS) for MM is about 4-5 years, but the OS is highly variable in different MM patients; some patients with aggressive disease courses may die in a few months after diagnosis, while other patients with indolent courses may live more than ten years [1]. Consequently, it is important to find a simple and robust stratification system to predict prognosis and help to optimize treatment strategy early in the course of myeloma [2].

Exploring a useful prognostic system has been a topic of interest in the myeloma filed since the past forty years, and considerable progresses have been made now. The Durie-Salmon system was established in 2005 and worldwide used since then. This system can divide patients into three stages by tumor burden [3]. However, this system is complicated and not objective. In 2005 the International Myeloma Working Group (IMWG) developed a new stage system called the International Staging System (ISS), which was relying on the combinations of two easily available and objective prognostic variables (serum beta 2 microglobulin (S*β*_2_M) and serum albumin): ISS stage I, S*β*_2_M less than 3.5 mg/L plus serum albumin ≥3.5 g/dL; ISS stage II, neither stage I nor stage III; and ISS stage III, S*β*_2_M ≥ 5.5 mg/L [4] (Table 1). The ISS system is the most important and commonly used stage system today. However, difficulties have been encountered now. When ISS system was defined, it contained data from patient with MM between 1981 and 2002. All of these patients were treated by conventional agents, not exposed to novel agents. However current treatment strategies for MM have been completely changed during the last decade by the introduction of novel agents. Novel agents such as bortezomib and thalidomide have become the most important component in MM therapy and dramatically improved the response rate, progression-free survival (PFS), and even OS of MM patients [5]. Thus the prognostic value of ISS system in the era of novel agents is still in debate.

Although recent studies had been focused on the applicability of ISS in the era of novel agents, until recently there was no conclusion. One study indicated that the ISS system was still robust after introduction of thalidomide in Greece [6]. A study from Dimopoulos et al. then demonstrated the applicability of ISS in MM patients with renal dysfunction [7]. Another study from Yang et al. indicates that ISS was not suitable for patients who underwent hematopoietic stem cell transplantation (HSCT) after the introduction of thalidomide [8]. Novel agents are well tolerated and have been recommended as the first-line choice at induction chemotherapy in National Comprehensive Cancer Network (NCCN), European Society for Medical Oncology (ESMO) guideline [9, 10]. The majority of patients received at least one kind of novel agents, such as bortezomib, thalidomide, or lenalidomide at induction therapy now. However, data is still less accurate for the prognostic significance of ISS in those who were acutely treated with novel agents at induction therapy in Chinese patients.

Therefore, we investigated a consecutive cohort of patients with MM who were treated with bortezomib- or thalidomide-based regimens as induction treatment in three Chinese centers, to validate the prognostic significance of the ISS in the era of novel agents in Chinese patients.

## 2. Methods

### 2.1. Patients and Treatment

A total of 1016 newly diagnosis symptomatic MM patients were enrolled between August 2006 and December 2012, from three Chinese myeloma centers (Department of Hematology at Changzheng Hospital, Peking University People's Hospital and Chaoyang Hospital). All of patients were diagnosed according to IMWG criteria [11]. The approvals were obtained from the Scientific Committee of three hospitals for the use of patients' medical records and publication of these data.

Patients who received at least two courses of one novel agent based therapy in the first-line treatment were included in this study. Thalidomide and bortezomib were introduced in the treatment of MM patients in China in 1999 and 2005, respectively, while lenalidomide was not available until 2013, before this analysis was conducted. The patients were divided into two groups by the type of the first-line regimens, bortezomib-based group and thalidomide-based group.

Patients' characteristics at diagnosis including gender, age, immunophenotype, ISS stage, peripheral neuropathy (PN), hemoglobin, platelets, bone marrow (BM) plasma cell percentage, serum calcium, serum albumin, S*β*_2_M, serum lactate dehydrogenase (LDH), and serum creatinine were collected.  Table 2 reported details regarding patients' clinical and hematological features.

### 2.2. Follow-Up

The last follow-up was conducted in March 2013. The primary end point for this study was OS, while secondary end points were PFS and response rate. OS was defined as the time between the diagnosis and death of any cause or until the last follow-up. PFS was defined as the time between the diagnosis and progression or until the last follow-up. Response rate to induction therapy was defined according to IMWG criteria. Patients were considered responsive when they achieved at least partial response (PR) in the first-line treatment.

### 2.3. Statistical Analysis

Statistical analyses were performed using SPSS version 18.0. Survival curves were plotted by using the Kaplan-Meier method. OS between the stages were tested using the log-rank test, with *p* < 0.05 taken as level of significance.

## 3. Results

### 3.1. Patient Characteristics

As showed in Table 2, 1016 patients with MM were enrolled in this study, 60.5% were male, the median age was 59 years, and the major subtypes were IgG (44.1%), IgA (22.2%), and light chain (23.1%). At diagnosis, 61.5% of patients had anemia (defined as hemoglobin <10 g/dL), 19.2% had renal dysfunction (defined as serum creatinine (Cr) ≥ 2 mg/mL), and 36.3% of patients' bone marrow plasma cell in filtration was more than 40%.

We divided 1016 patients into two groups by the types of novel agents in the first-line treatment, 709 patients in bortezomib-based group (defined as at least received 2 cycles of bortezomib-based treatment in first-line treatment) and 307 patients in thalidomide-based group (defined as at least received 2 cycles of thalidomide-based treatment in first-line treatment). The regimens of first-line therapy in each group were also listed in Table 2. The number of patients exposed to both bortezomib and thalidomide in our database is too small (41 patients). Thus the data of these patients were not included in this study. Compared to patients in thalidomide-based group, patients in bortezomib-based group had more elder patients (*p* = 0.0001), higher BM plasma cell percentage (*p* = 0.012), and better response rate (*p* < 0.0001).

### 3.2. Patient Outcome in the Entire Cohort

**Figure 1:**
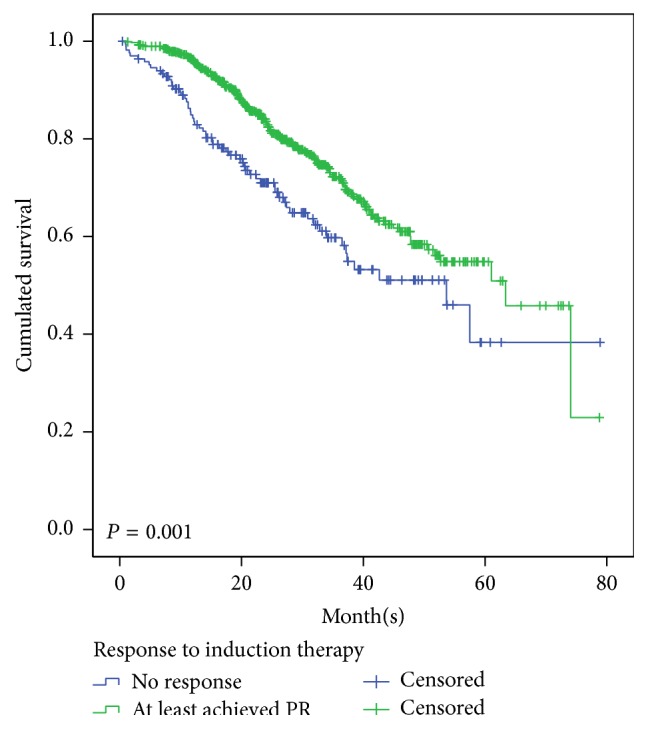
Overall survival (OS) for 1016 patients according to the response to the first-line therapy who at least achieved PR and who were below PR.

The median estimated follow-up for the cohort was 24.3 months with 72.1% alive at last follow-up. The median OS was 55.8 months and PFS was 28.0 months for the entire patients. In this study, it showed a significant better survival in patients who at least achieve PR in the first-line therapy than those who did not, median OS was 63.4 versus 53.7 months, and 5-year survival was 50.9% versus 38.3% (*p* < 0.0001) (Figure 1).

### 3.3. Validate Prognostic Value of ISS System in the Entire Cohort

**Figure 2:**
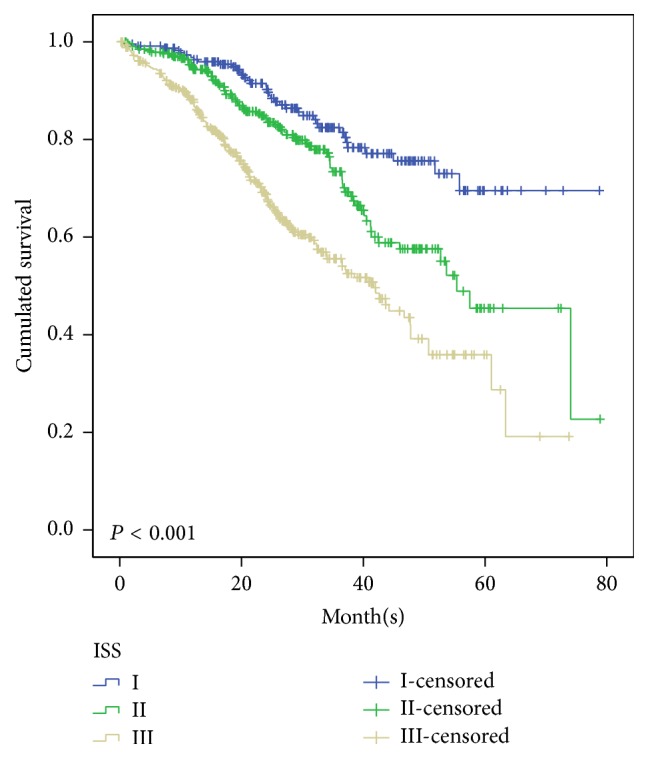
Overall survival for 1016 patients with newly diagnosed symptomatic multiple myeloma according to the ISS stages I, II, and III, who were treated with novel agents as the first-line strategy.

The prognostic value of ISS system was evaluated in the total population of 1,016 patients. Patients were divided into stages I/II/III according to ISS system, and corresponding proportion in each stage was 22.7%/33.6%/43.7%, respectively. The median OS for ISS stages I/II/III was not reached/55.4 months/41.7 months (Figure 2), and the median PFS was 30/29.5/25 months (*p* = 0.072), respectively. From these data, we can conclude that ISS system can predict prognosis for OS, but for PFS in MM patients in the entire cohort.

### 3.4. Validate Prognostic Value of ISS System in Bortezomib-Based or Thalidomide-Based Group

In order to discern the impact of novel agents to the ISS system, subgroup analyses for OS were also performed in patients who received bortezomib-based treatment and thalidomide-based treatment in the first-line therapy.

**Figure 3:**
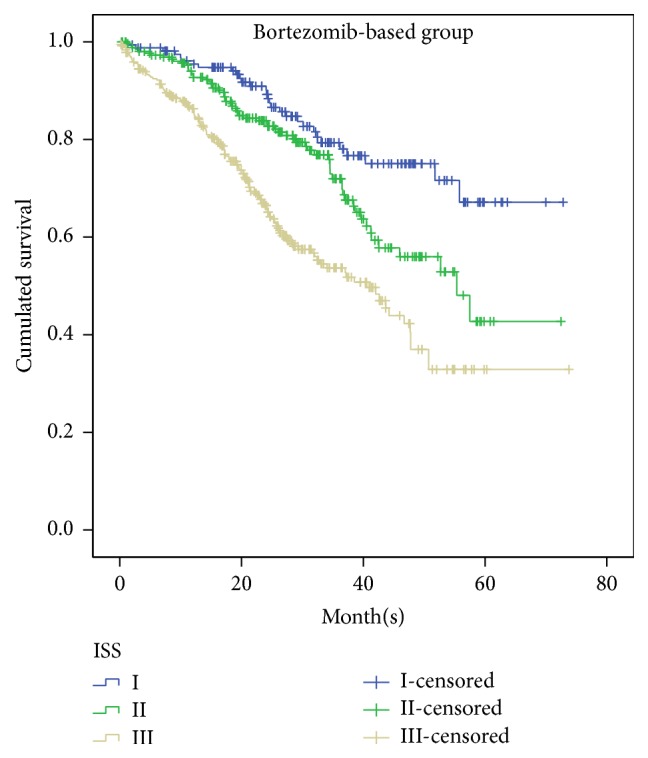
Overall survival for 709 patients with newly diagnosed symptomatic multiple myeloma according to the ISS stages I, II, and III, who were treated with bortezomib-based treatment as first-line therapy.

In bortezomib-based group, 170 patients were in ISS-I, 236 in ISS-II, and 303 in ISS-III, with median OS being not reached/57.5 months/42.0 months and 3-year survival was 77.7%/75.8%/53.4%, respectively (Figure 3). Statistical difference was verified between ISS-I and ISS-III (*p* < 0.0001), ISS-II and ISS-III (*p* < 0.0001), and ISS-I and ISS-II (*p* = 0.038). In order to validate ISS system in transplant and nontransplant patients, we further stratified bortezomib-based patients into two subgroups according to whether they underwent transplantation after induction therapy. In bortezomib-based group, 177 patients underwent transplantation and 532 patients did not, the median OS was 51.8 months versus 57.5 months, respectively. The 3-year survival was 76.1% versus 69.2% versus 56.2% in nontransplantation group and 89.7% versus 86.7% versus 42.3% in transplantation group, respectively. The similar result was showed in transplantation and nontransplantation group, and no statistical difference was showed between ISS-I and ISS-II in bortezomib-based transplantation (*p* = 0.413) and nontransplantation groups (*p* = 0.056).

**Figure 4:**
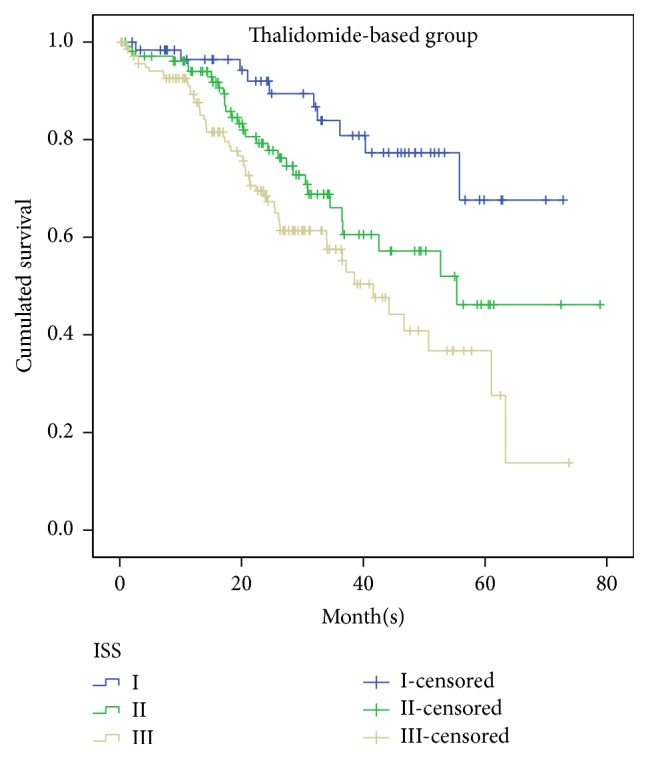
Overall survival for 307 patients with newly diagnosed symptomatic multiple myeloma according to the ISS stages I, II, and III, who were treated with thalidomide-based treatment as first-line therapy.

In thalidomide-based group, 61 patients were classified in ISS-I, 105 in ISS-II, and 141 in ISS-III, with median OS being not reached, 55.4 months, and 41.7 months, respectively (Figure 4). Statistically significant difference in survival was confirmed between three stages, ISS-I and ISS-II (*p* = 0.024), ISS-I and ISS-III (*p* < 0.0001), and ISS-II and ISS-III (*p* = 0.047), respectively. Among these patients, 282 patients underwent transplantation and 25 patients did not. Because the number in transplantation group is quite small, we do not carry out analysis in transplant group. In nontransplant group, 54 patients were in ISS-I, 95 in ISS-II, and 133 in ISS-III. There was statistical difference between ISS-I and ISS-II (*p* = 0.009) and ISS-I and ISS-III (*p* < 0.0001), but no statistical difference between ISS-II and ISS-III (*p* = 0.089). Although ISS prognostic significance disappeared between ISS-II and ISS-III in thalidomide-based nontransplant group, this may be due to the small number in ISS stages II and III; more studies are needed in the future.

## 4. Discussion

Multiple myeloma (MM) is characterized by heterogeneity in the clinical course and risk stratification is vital for prediction of prognosis. ISS is the most important prognostic system for MM in the past ten years. This system predicts survival of newly diagnosed MM patients by using two routine and inexpensive pieces of laboratory data and separated patients into three stages with a distinct prognosis [4]. Although ISS system was wildly used in Chinese myeloma patients in the past decade, the original analysis of ISS system from Greipp et al. did not include Chinese patients' data. Besides these, the survival of MM has dramatically changed by the introduction of novel agents, and nowadays the majority of patients received novel agent based treatment in the first-line therapy. Thus, the initial question that motivates our study was to determine whether ISS is suitable in the era of novel agents and in Chinese MM patients. This study aimed to provide outcome data for patients actually exposed to novel agents at first-line treatment.

In this analysis, we enrolled consecutive patients; thus the results may be more appropriate to the general group of myeloma patients, for patients enrolled in clinical trials were selected by some screening conditions. In this study, all of patients exposed to novel agents in first-line therapy. Survival in patients who achieved at least PR at induction therapy is much better than those who did not responsd, due to better outcome with more aggressive therapy. These results confirmed the Dimopoulos et al.'s observations [12].

The ISS system still has prognostic significance value when applied to the total 1016 patients, with median OS for ISS stages I/II/III being not reached/55.4 months/41.7 months (*p* < 0.001). When ISS was proposed the median OS of MM patients in ISS stages I/II/III was 64, 44, and 29 months, respectively. Thus this study indicates that the survival of patients in each ISS stage is significantly improved in the era of novel agents. This significance partly disappeared when ISS implied to patients who received bortezomib-based regimens in the first-line treatment. We demonstrated the ISS system still has prognostic value in the era of novel agents in Chinese patients with MM, while in subgroup analysis it is not fully applicable and limited prognostic value in patients receiving bortezomib-based treatment in first-line therapy.

There is few data on the applicability of ISS in bortezomib-based treatment in the first-line therapy in literature. From previous studies we can indicate that MM patients can achieve deeper response by the use of novel agents, improved PFS and OS [13]. A meta-analysis performed by Zou et al. showed the addition of bortezomib to first-line therapy did significantly prolong OS compared with conventional therapy alone [14]. Some studies have showed that bortezomib-based regimens can improve outcome of patients with t(4;14), deletion of chromosome 13, and deletion of 17p, respectively [15–17]. This study also figured out patients with either t(4;14) or del(17p) presented in a higher ISS stage. We can find out that bortezomib has shown survival benefit in myeloma and overcome specific cytogenetic risk features in MM patients. This may partly explain in our study no statistical difference in OS between ISS stages I and II.

The ISS system was used as an independent prognostic system in the past, but it was unable to reflect the cytogenetic abnormalities of MM. Some new prognostic factors were increasingly found, such as fluorescent in situ hybridization (FISH), karyotype, and serum-free light chain [18–21]. These new prognostic factors can overcome this deficiency and provide cytogenetic or molecular genetics-based risk classification for MM patients. Many efforts have been made, such as proposing a new stage system by combination of ISS with FISH [22]. A recent study from IMWG combined ISS, CA, and LDH data to define Revised International Staging System (R-ISS) by following three risk categories: R-ISS I including ISS stage I, no high-risk CA [del(17p) and/or t(4;14) and/or t(14;16)], and normal LDH level; R-ISS III including ISS stage III and high-risk CA or high LDH level; and R-ISS II including all the other combinations. The data of R-ISS were enrolled on 11 clinical trials from 2005 to 2013. All patients received new drugs based chemotherapy as up-front treatment. The 5-year OS rate in R-ISS I, II, and III was 82%, 62%, and 40%, respectively. In our data, few patients had the data of chromosomal abnormalities (CA) detected by interphase fluorescent in situ hybridization after CD138 plasma cell purification. Compared with these IMWG studies, the majority of patients were in intermediate-risk group; in our study, 43.7% of patients were in high-risk group (ISS stage III). This distribution may explain worse survival in our study in three stage groups. The R-ISS system can predict prognosis on OS in patients who did receive proteasome inhibitor based treatment, while in our study the ISS system cannot clearly distribute the OS of MM patients in ISS stages I and II. One interpretation might be that, compared with R-ISS system, ISS system may wrongly allocate a certain group of patients with poor prognosis in lower ISS stage [23].

There are many restrictive conditions for these new variables, for example, no consensus in standard classifications, not being easily available, and being too expensive. Their applications were limited by these passive factors. Thus, although novel prognostic factors such as FISH, karyotype, and serum-free light chain are important in MM risk stratification, the prognostic value of traditional serum markers still deserves attention. It can be an important component of new staging system in the future. Reevaluating the prognostic value of ISS system now is beneficial for the future research for a new staging system.

Because the initial retrospective study design from which these data are obtained was focused on clinical features and outcome in Chinese patients, the data analysis was presented with a number of challenges including (1) inconsistencies in patients feature among two groups, (2) the inability to study the effect of novel agents by using the same regimens in each group, and (3) needing a very long follow-up time and a very large patients' series to prove OS benefit in the era of new agents. Unfortunately, we could not evaluate the impact of adverse genetic markers in our cohort of patients because FISH studies were performed in a minority of patients.

## 5. Conclusion

In conclusion, our data is the first multicenter retrospective study in Chinese myeloma patients that validates ISS value in a large number of unselected patients. The results demonstrated that International Staging System still has prognostic value in the era of novel agents in Chinese patients with MM. However, that ISS system is not fully applicable in patients receiving bortezomib-based therapy at first-line treatment. These findings suggest that ISS system is predictive for OS of Chinese MM patients in the era of novel agents, but value is limited in PFS and in patients who were exposed to bortezomib in the first-line therapy. Bortezomib may have the potential to partially overcome adverse effect of risk factors on survival, especially in higher stage of ISS system. Further study is needed to develop more suitable staging system applied to MM patients in the era of novel agents which can reflect not only tumor burden and patient's condition, but also genetic risk classification.

## Figures and Tables

**Table 1:** The definition of the ISS system.

ISS stage	Definition	Median OS
Stage I	S*β*_2_M less than 3.5 mg/L plus serum albumin ≥3.5 g/dL	64 months
Stage II	Neither stage I nor stage III	44 months
Stage III	S*β*_2_M ≥5.5 mg/L	29 months

ISS: International Staging System, OS: overall survival.

S*β*_2_M: serum beta 2 microglobulin.

**Table 2:** Patient characteristics at diagnosis according to the type of novel agents in the first-line therapy.

Parameters	Total (%)	Bortezomib-based group (%)	Thalidomide-based group (%)	*p* value
Patient (*n*)	1016	709	307	
Male	59.7%	60.1%	59.0%	0.394
Age >60 years	44.2%	40.3%	53.1%	0.0001
Hemoglobin <10 g/dL	61.5%	60.2%	64.8%	0.253
Creatinine ≥2 mg/mL	19.2%	20.3%	16.4%	0.183
BM plasma cell percentage ≥40%	36.3%	38.8%	30.2%	0.012
Platelet counts <130 *∗* 10^9^/L	30.9%	31.7%	29.2%	0.454
PN	12.3%	10.7%	15.4%	0.063
LDH ≥245 U/L	14.6%	14.9%	13.8%	0.689
Albumin (<35 g/L)	47.4%	43.6%	56.1%	0.0001
*β*_2_-MG (≥3.5 mg/L)	55.3%	56.1%	53.4%	0.028
DS stage				0.483
I	2.5%	2.2%	3.6%	
II	9.5%	9.3%	10.1%	
III	88.0%	88.5%	86.3%	
ISS stage				0.343
I	22.7%	24.0%	26.4%	
II	33.6%	33.3%	34.2%	
III	43.7%	42.7%	45.9%	
Myeloma type				0.050
IgG	44.1%	42.6%	47.6%	
IgA	22.2%	23.6%	19.2%	
IgD	7.4%	8.5%	4.9%	
*κ* light chain	12.0%	12.1%	11.7%	
*λ* light chain	11.1%	11.0%	11.4%	
others	3.2%	2.2%	5.2%	
Regimens of the first-line therapy		PAD/VD/BCD/V-DECP	TAD/TD/MPT/CTP/T-DECP	
≥PR to the first-line therapy	80%	84.1%	68.8%	0.0001

BM: bone marrow, PN: peripheral neuropathy, LDH: lactate dehydrogenase, DS stage: Durie-Salmon stage, ISS: International Staging System, OS: overall survival, PAD: Bortezomib (Velcade), Adriamycin, and Dexamethasone, VD: Bortezomib (Velcade) and Dexamethasone, BCD: Bortezomib (Velcade), Cyclophosphamide, and Dexamethasone, V-DECP: Bortezomib Cisplatin, Etoposide, Cyclophosphamide, and Dexamethasone; TD: Thalidomide and Dexamethasone, TAD: Thalidomide, Adriamycin, and Dexamethasone, MPT: Melphalan, Prednisone, and Thalidomide, T-DECP: Thalidomide, Cisplatin, Etoposide, Cyclophosphamide, and Dexamethasone; CTP: Cyclophosphamide, Thalidomide, and Dexamethasone, and PR: partial response.
